# Supplementary material for: Computational and neural mechanisms of statistical pain learning
Source: Nat Commun. 2022 Nov 3;13:6613. doi: 10.1038/s41467-022-34283-9 (PMC9633765; doi:10.1038/s41467-022-34283-9)
Supplement: Supplementary file 1 — Supplementary Information [file 41467_2022_34283_MOESM1_ESM.pdf]

Computational and neural mechanisms of statistical pain learning - Supplementary Information

SUPPLEMENTARY NOTE 1

To carry out model's recovery, we simulated each model for 10 values evenly spaced from the minimum to maximum parameter range fitted to participant data, using a stimulus sequence and a random selection of trials with responses from different pilot participants. Then, we fitted each model to its own data to minimize BIC. We found a high correlation between simulated and fitted parameters for all models except the random model (Supplementary Table 1), which indicates a successful recovery of the model parameters.

Model	Rho
Bayesian Jump Freq	0.996
Bayesian Jump TP	0.991
Bayesian Fixed Freq	0.942
Bayesian Fixed TP	0.880
Rescorla-Wagner	0.986
Random	0.468

Supplementary Table 1. Pearson's correlation coefficients between simulated and fitted parameters

SUPPLEMENTARY NOTE 2

To inspect the quality of fit of the winning model (Bayesian jump frequency model), we evaluated the relation between the frequency p(H) predicted by the Bayesian jump frequency model vs. individual participants (one regression line for each participant in Supplementary Figure 1). At group level, the correlation coefficients were significantly above 0 ($r=0.829$, $t(35)=8.629$, $p<0.001$, two-sided statistics).

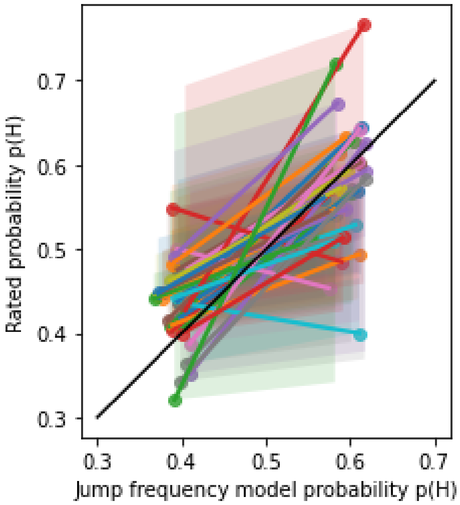

Supplementary Figure 1. Frequency of high pain predicted by the Bayesian jump frequency model vs. human participants. One regression line per participant. The black line is shown only for reference.

.

SUPPLEMENTARY NOTE 3

We evaluated, in each participant, the correlation between the high pain rating (averaged across blocks) and the prediction accuracy (measured by the correlation coefficient between rated and true frequencies and transition probabilities). There was no evidence for a correlation between mean pain intensity and prediction accuracy (frequency prediction accuracy by high pain intensity: r = -0.175, p = 0.337; p(H | H) prediction accuracy by high pain intensity: r = -0.178, p = 0.305; 2).

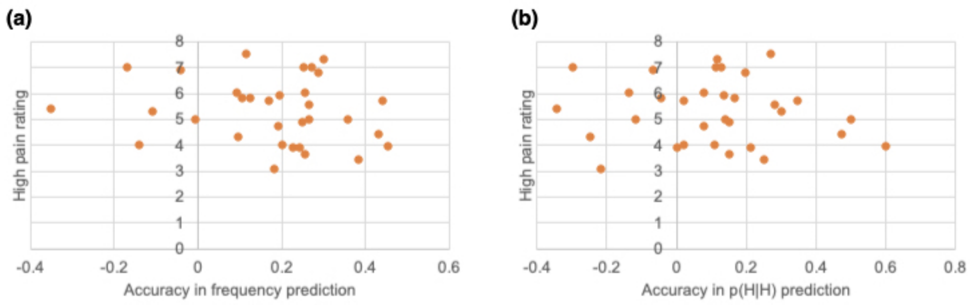

Supplementary Figure 2. Mean intensity of high stimulus by prediction accuracy for frequencies (a) and transition probabilities (b).

.

SUPPLEMENTARY NOTE 4

	Cluster ID	X	Y	Z	Peak Stat	Cluster Size (mm3)
H > L						
0	1	13	-17	5	6.886	2587
1	1a	13	-22	-3	4.957	
2	2	33	-22	58	6.524	5247
3	2a	30	-19	49	5.953	
4	2b	16	-17	68	5.524	
5	3	37	-17	15	5.742	1186
6	4	6	-7	49	5.128	3486
7	4a	0	-2	43	4.967	
8	4b	4	-19	49	4.333	
9	4c	11	-14	49	4.289	
10	5	-17	-60	-19	4.851	1707
11	5a	-10	-55	-16	4.651	
12	5b	-7	-62	-22	4.217	
H < L						
0	1	47	-7	30	7.211	6218
1	1a	61	-7	30	7.067	
2	1b	40	-26	40	4.455	
3	1c	47	-19	43	4.453	
4	2	-60	-7	33	6.263	12669
5	2a	-67	-10	24	5.806	
6	2b	-53	-10	43	5.170	
7	2c	-26	-41	49	5.074	
8	3	4	-38	61	5.249	2354
9	4	-22	-79	36	5.231	1006

Supplementary Table 2. Activation clusters for the High > Low pain and High < Low pain contrasts (FDR corrected $p<0.001$).

SUPPLEMENTARY NOTE 5

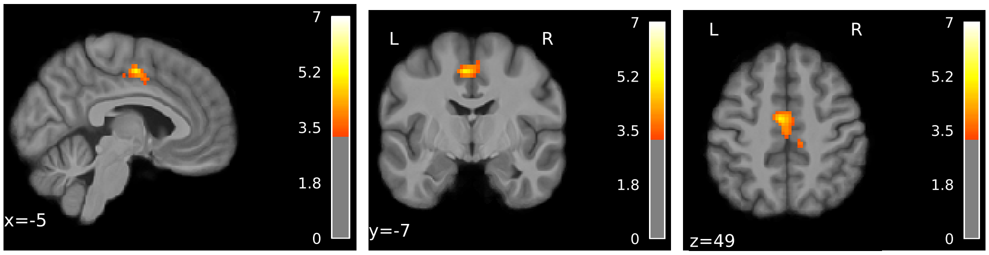

(a) **(b)** **(c)**

Supplementary Figure 3. Negative contrast of the KL divergence in the Bayesian jump frequency model, in all subjects. The z-statistics, shown in the colorbar, has been FDR corrected p<0.001 and thresholded at Z>3.3. The images are shown in sagittal (a), coronal (b) and axial (c) views.

	Cluster ID	X	Y	Z	Peak Stat	Cluster Size (mm3)
0	1	-5	-7	49	5.096	3414
1	1a	9	-19	43	4.766	
2	1b	0	-17	46	4.513	

Supplementary Table 3. Bayesian model (jump frequency) KL divergence (negative contrast) activation clusters

SUPPLEMENTARY NOTE 6

We conducted three separate fMRI analyses, each adding a different covariate to the generalised linear mixed model presented in the main article (regressors of interest: posterior mean p(high), SD posterior, KL divergence, stimulus intensity). The covariates we tested were:

(1) the evidence of the Bayesian jump frequency model;
(2) the prediction accuracy, as indexed by the coefficient of the correlation between the transition probability p(L | H) rated by the subject and the true p(L | H);
(3) an alternative measure of prediction accuracy, i.e. the coefficient of the correlation between the stimulus frequency predicted by the subject vs. the Bayesian jump frequency model.

The neural correlates of the mean posterior of low/high pain (probabilistic inference), model update and posterior SD (uncertainty of the inference) were very similar across the different control analyses we conducted (figures 4, 5, 6), and also remarkably similar to those reported in the main article (figures 9-12).

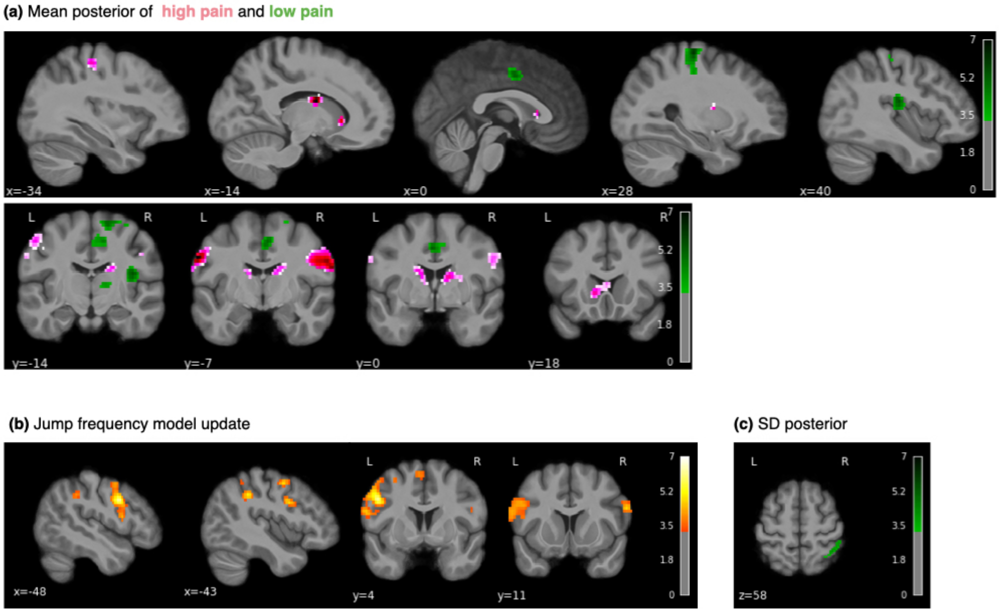

Supplementary Figure 4. Neural correlates of the (a) mean posterior of low (green) and high (pink) pain frequency, (b) update of the Bayesian jump frequency model, and (c) uncertainty after controlling for model evidence.

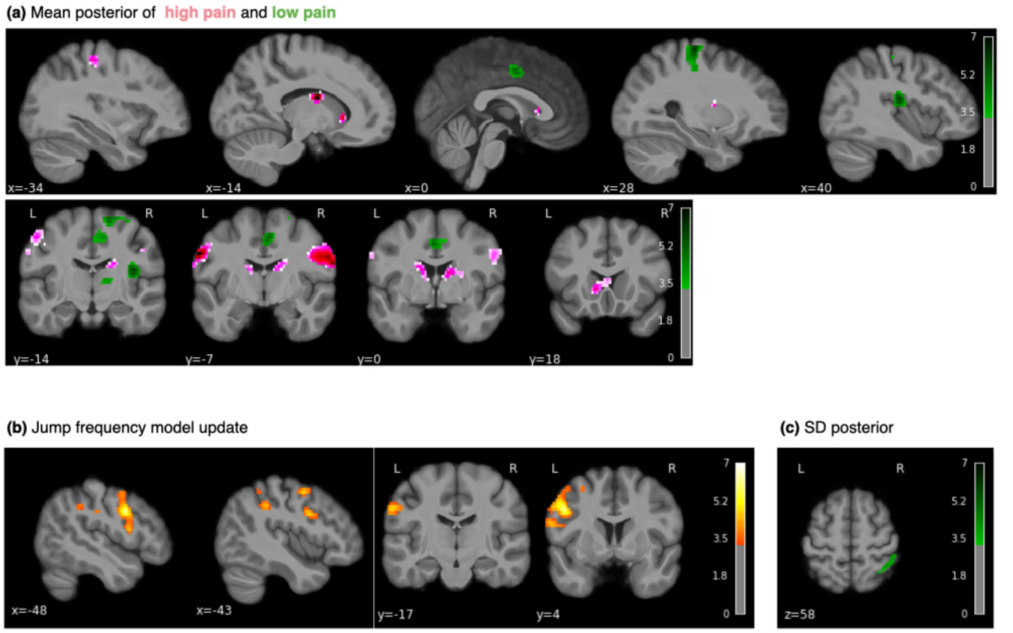

Supplementary Figure 5. Neural correlates of the (a) mean posterior of low (green) and high (pink) pain frequency, (b) update of the Bayesian jump frequency model, and (c) uncertainty after controlling for prediction accuracy (i.e. the coefficient of the correlation between the transition probability p(L | H) rated by the subject and the true (generative) p(L | H).

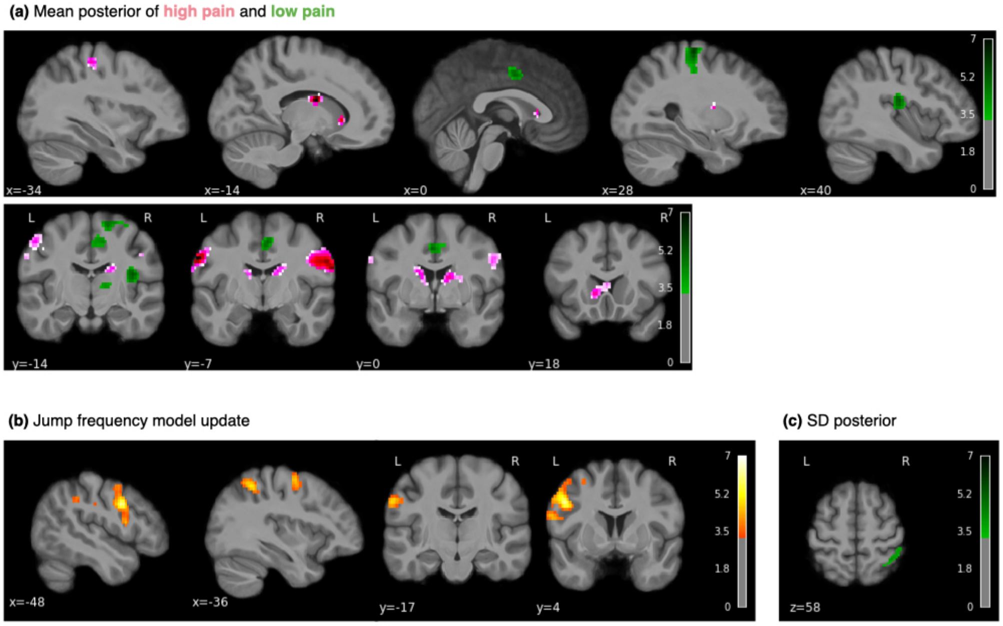

Supplementary Figure 6. Neural correlates of the (a) mean posterior of low (green) and high (pink) pain frequency, (b) update of the Bayesian jump frequency model, and (c) uncertainty after controlling for an alternative measure of prediction accuracy (i.e., the correlation between the frequency of low pain rated by the subject and predicted by the Bayesian jump frequency model.

SUPPLEMENTARY NOTE 7

Our model fitting and comparison analyses indicate that participants in our sample used a Bayesian inference strategy with dynamic update of beliefs, but there were inter-individual differences in the nature of temporal statistics inferred. Whereas 23 participants favoured the inference of the frequency of the stimuli, 12 participants preferred to infer the transition probability (TP) of the stimuli, which yields more accurate predictions. Thus, we conducted additional follow-up neuroimaging analyses to explore these group differences.

First, we divided participants in two groups, according to their preferred inference strategy, defined as the model with the highest evidence (frequency: n=23, TP: n=12). For each subject, we derived the mean posterior inference, SD posterior and model update (KL divergence between two consecutive posterior distributions) in the jump frequency model and in the jump TP model. After convolving them with a hemodynamic response function, we used them as trial-by-trial regressors for BOLD responses, on each individual, separately for the two models. We then contrasted neural correlates of inference, uncertainty and model update between the two groups (preferred learning strategy: frequency vs. TP), separately for each model (Bayesian jump frequency vs. TP).

We found no significant group differences in the neural correlates of predictive inference (mean posterior of stimulus frequency) and uncertainty (SD posterior of stimulus frequency) in either model. However, the update of the jump frequency and TP models (KL divergence) was associated with increased activity in the left orbitofrontal cortex in the group that favoured frequency inference than in the group that favoured TP inference (jump frequency model: peak x -24, y 28, z -16, z-stat 4.806; jump TP model: peak x -26, y 25, z -19, z-stat 4.504; Supplementary Figure 7).

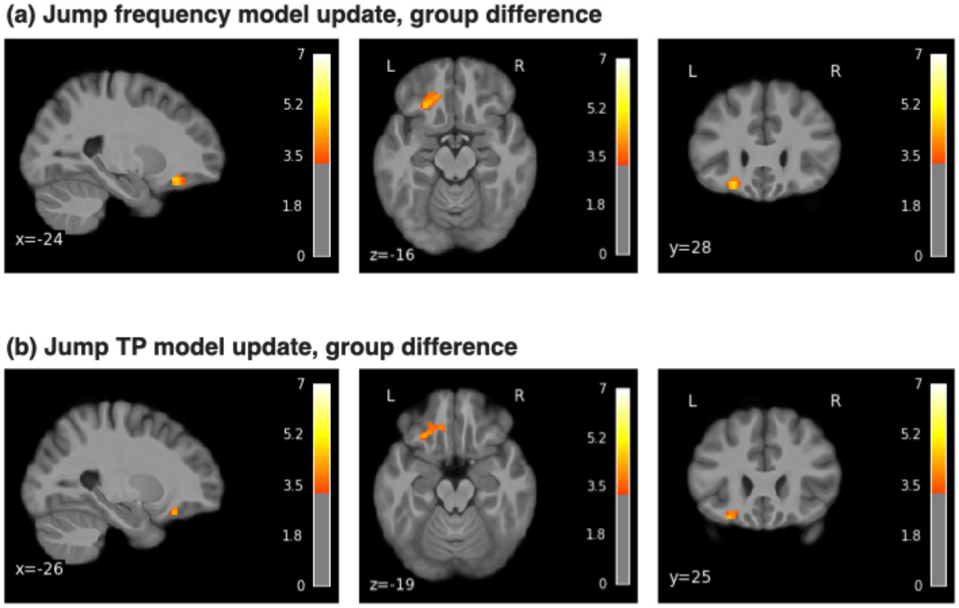

Supplementary Figure 7. The left orbitofrontal cortex was more active in the group of participants that favoured a frequency inference strategy over a transition probability strategy, in association with the update of the jump frequency (a) and transition probability models (b).
