# Supplementary material for: Computational and neural mechanisms of statistical pain learning
Source: Nat Commun. 2022 Nov 3;13:6613. doi: 10.1038/s41467-022-34283-9 (PMC9633765; doi:10.1038/s41467-022-34283-9)
Supplement: Supplementary file 3 — Reporting Summary [file 41467_2022_34283_MOESM3_ESM.pdf]

nature portfolio

Corresponding author(s): Flavia Mancini

Last updated by author(s): Oct 14, 2021

Reporting Summary

Nature Portfolio wishes to improve the reproducibility of the work that we publish. This form provides structure for consistency and transparency in reporting. For further information on Nature Portfolio policies, see our Editorial Policies and the Editorial Policy Checklist.

Statistics

For all statistical analyses, confirm that the following items are present in the figure legend, table legend, main text, or Methods section.

n/a	Confirmed	
☐	☒	The exact sample size (*n*) for each experimental group/condition, given as a discrete number and unit of measurement
☐	☒	A statement on whether measurements were taken from distinct samples or whether the same sample was measured repeatedly
☐	☒	The statistical test(s) used AND whether they are one- or two-sided<br>*Only common tests should be described solely by name; describe more complex techniques in the Methods section.*
☐	☒	A description of all covariates tested
☐	☒	A description of any assumptions or corrections, such as tests of normality and adjustment for multiple comparisons
☐	☒	A full description of the statistical parameters including central tendency (e.g. means) or other basic estimates (e.g. regression coefficient) AND variation (e.g. standard deviation) or associated estimates of uncertainty (e.g. confidence intervals)
☐	☒	For null hypothesis testing, the test statistic (e.g. F, t, r) with confidence intervals, effect sizes, degrees of freedom and P value noted<br>*Give P values as exact values whenever suitable.*
☐	☒	For Bayesian analysis, information on the choice of priors and Markov chain Monte Carlo settings
☐	☒	For hierarchical and complex designs, identification of the appropriate level for tests and full reporting of outcomes
☐	☒	Estimates of effect sizes (e.g. Cohen's d, Pearson's r), indicating how they were calculated

Our web collection on statistics for biologists contains articles on many of the points above.

Software and code

Policy information about availability of computer code

Data collection	https://doi.org/10.5281/zenodo.6997897
Data analysis	https://doi.org/10.5281/zenodo.6997897, Imaging data were preprocessed using fmriprep (pypi version: 20.1.1, RRID:SCR_016216) with Freesurfer option disabled, within its Docker container. Processed functional images had first four dummy scans removed, and then smoothed in an 8mm Gaussian filter in SPM12. Nipype (pypi version: 1.5.1) was used for all fMRI processing and analysis within its published Docker container.

For manuscripts utilizing custom algorithms or software that are central to the research but not yet described in published literature, software must be made available to editors and reviewers. We strongly encourage code deposition in a community repository (e.g. GitHub). See the Nature Portfolio guidelines for submitting code & software for further information.

Data

Policy information about availability of data

All manuscripts must include a data availability statement. This statement should provide the following information, where applicable:

- Accession codes, unique identifiers, or web links for publicly available datasets
- A description of any restrictions on data availability
- For clinical datasets or third party data, please ensure that the statement adheres to our policy

Raw functional imaging data is deposited at OpenNEURO doi:10.18112/openneuro.ds003836.v1.0.0 and derived statistical maps are available at NeuroVault (https://neurovault.org/collections/12827/). Sequence generation, task instructions and behavioural data can be found at https://doi.org/10.5281/zenodo.6997897

Field-specific reporting

Please select the one below that is the best fit for your research. If you are not sure, read the appropriate sections before making your selection.

☐ Life sciences ☒ Behavioural & social sciences ☐ Ecological, evolutionary & environmental sciences

For a reference copy of the document with all sections, see nature.com/documents/nr-reporting-summary-flat.pdf

Behavioural & social sciences study design

All studies must disclose on these points even when the disclosure is negative.

Study description	Quantitative experimental: temporal statistical learning task
Research sample	35 healthy participants (17 females; mean age 27.4 years old; age range 18-45 years) took part in the study comprising two experimental sessions. Each participant gave informed consent according to procedures approved by University of Cambridge ethics committee (PRE.2018.046). The sample size was chosen based on a previous study with visual stimuli.
Sampling strategy	Random sampling of volunteers. Sample size was determined based on a previous study using visual stimuli (Meyniel 2016).
Data collection	Computer running matlab code was used to generate the stimulus sequence and collect responses. In the scanner, the participant responded using a response box, and data were automatically saved by matlab in a data file. The researcher was blinded to the stimuli that were delivered automatically by the computer. A radiographer was also present during MRI data acquisition.
Timing	Data collection happened between Dec 2018 and May 2019
Data exclusions	Pilot data (subjects 1-5) were excluded from analyses and not included in the calculation of the sample size. Subjects who did not complete the study were excluded from analyses. No other data were excluded.
Non-participation	One subject dropped out before the fMRI session, without providing a justification. This subject was excluded from the research sample.
Randomization	There were no groups, all participants performed the same task.

Reporting for specific materials, systems and methods

We require information from authors about some types of materials, experimental systems and methods used in many studies. Here, indicate whether each material, system or method listed is relevant to your study. If you are not sure if a list item applies to your research, read the appropriate section before selecting a response.

Materials & experimental systems

n/a	Involved in the study	
☒	☐	Antibodies
☒	☐	Eukaryotic cell lines
☒	☐	Palaeontology and archaeology
☒	☐	Animals and other organisms
☐	☒	Human research participants
☒	☐	Clinical data
☒	☐	Dual use research of concern

Methods

n/a	Involved in the study	
☒	☐	ChIP-seq
☒	☐	Flow cytometry
☐	☒	MRI-based neuroimaging

Human research participants

Policy information about studies involving human research participants

Population characteristics	35 healthy participants (17 females; mean age 27.4 years old; age range 18-45 years)
Recruitment	Participants signed up to the study advertised online, in our research studies website. MRI eligibility was checked in all participants. Typically, subjects who volunteer to take part in paid studies are younger or have lower social economical status. This is not expected to impact the results.
Ethics oversight	University of Cambridge ethics committee (PRE.2018.046)

Note that full information on the approval of the study protocol must also be provided in the manuscript.

Magnetic resonance imaging

Experimental design

Design type	Task, event-related design
Design specifications	The electrical stimuli were generated using a DS5 isolated bipolar current stimulator (Digitimer), delivered to surface electrodes placed on the index and middle fingers of the left hand. All participants underwent a standardised intensity work-up procedure at the start of each testing day, in order to match subjective pain levels across sessions to a low-intensity level (just above pain detection threshold) and a high-intensity level that was reported to be painful but bearable (>4 out of 10 on a VAS ranging from 0 ['no pain'] to 10 ['worst imaginable pain']). The pain delivery setup was identical for lab-based and MR sessions. After identifying appropriate intensity levels, we checked that discrimination accuracy was >95\% in a short sequence of 20 randomised stimuli. This was done to unsure that uncertainty in the sequence task would derive from the temporal order of the stimuli rather than their current intensity level or discriminability. If needed, we tweaked the stimulus intensities to achieve our target discriminability.<br>Next, we gave the task instructions to each participants.<br><br>After receiving a shock on trial t, subjects were asked to predict the probability of receiving a stimulus of the same or different intensity on the upcoming trial (trial t+1). We informed participants that in the task they "would receive two kinds of stimuli, a low intensity shock and a high intensity shock. The L and H stimuli would be presented in a sequence, in an order set by the computer. After each stimulus, the following stimulus could be either the same or different than the previous one. The computer sets the probability that after a given stimulus (for example L) there would be either L or H" (we showed a visual representation of this example). We asked participants to "always try to guess the probability that after each stimulus there will the same or a different one" and we informed them that "the computer sometimes changes its settings and sets new probabilities", so to pay attention all the time. We also told them the sequence would be paused occasionally in order to collect probability estimates from participants using the scale depicted in Fig \ref{fig1}. A white fixation cross was displayed on a dark screen throughout the trial, except when a response was requested every 12-18 trials. The interstimulus interval was 2.8-3 seconds. There were 300 stimuli in each block, lasting approx. 8 minutes. Average intensity ratings for each stimulus level were collected after each block during a short break. Low intensity stimuli were felt by participants as barely painful, rated on average 1.39 (SD 0.77) on a scale ranging from 0 (no pain) to 10 (worst pain imaginable). In contrast, high intensity stimuli were rated as more than 4 times higher than low intensity stimuli (mean 5.74, SD 4.85). Participants were given 4 blocks of practice, 2-3 days prior the imaging sessions, and 5 blocks (1500 stimuli in total) during task fMRI.<br><br>The sequence of stimuli was unique and generated as in Meyniel 2016 Plos Biol. L and H stimuli were drawn randomly from a 2x2 transition probability matrix, which remained constant for a number of trials (chunks). The probability of a change was 0.014. Chunks had to be >5 and <200 trials long. In each chunk, transition probabilities were sampled independently and uniformly in the 0.15–0.85 range (in steps of 0.05), with the constraint that at least one of the two transition probabilities must be >/< 0.2 than in the previous chunk. Participants were not informed when the matrix was resampled, and a new chunk started.
Behavioral performance measures	Participants rated the probability of forthcoming stimuli. We used conputational modelling to determine the underlying learning model, as described in "Methods/Computational modelling of temporal statistical learning"

Acquisition

Imaging type(s)	functional and structural
Field strength	3T
Sequence & imaging parameters	First, we collected a T1-weighted MPRAGE structural scan (voxel size 1 mm isotropic) on a 3T Siemens Magnetom Skyra (Siemens Healthcare), equipped with a 32-channel head coil (Wolfson Brain Imaging Centre, Cambridge). Then we collected 5 task fMRI sessions of 246 volumes using a gradient echo planar imaging (EPI) sequence (TR = 2000 ms, TE = 23 ms, flip angle = 78°, slices per volume = 31, Grappa 2, voxel size 2.4 mm isotropic, A>P phase-encoding; this included four dummy volumes, in addition to those pre-discarded by the scanner). In order to correct for inhomogeneities in the static magnetic field, we imaged 4 volumes using an EPI sequence identical to that used in task fMRI, inverted in the posterior-to-anterior phase encoding direction. Full sequence metadata are available at OpenNeuro (https://openneuro.org/datasets/ds003836).
Area of acquisition	whole brain

Diffusion MRI ☐ Used ☒ Not used

Preprocessing

Preprocessing software	Imaging data were preprocessed using fmriprep (pypi version: 20.1.1, RRID:SCR_016216) with Freesurfer option disabled, within its Docker container. Processed functional images had first four dummy scans removed, and then smoothed in an 8mm Gaussian filter in SPM12.
Normalization	fmriprep runs spatial normalization as part of the preprocessing, which normalized images standard MNI spaces by using ANTs' antsRegistration in a multiscale, mutual-information based, nonlinear registration scheme.

Normalization template	MNI152NLin2009cAsym was used as the standard space reference template, as the default setting of fmriprep.
Noise and artifact removal	Nipype (pypi version: 1.5.1) was used for all fMRI processing and analysis within its published Docker container. Nipype is a python package that wraps around fMRI analysis tools including SPM12 and FLS in a Debian environment.<br><br>First and second level GLM analyses were conducted using SPM12 through nipype. In all first level analyses, 25 regressors of no interest were included from fmriprep confounds output: CSF, white matter, global signal, dvars, std_dvars, framewise displacement, rmsd, 6 a_comp_cor with corresponding cosine components, translation in 3 axis and rotation in 3 axis. Sessions within subject are not concatenated.
Volume censoring	we used the defaults of the fmriprep package

Statistical modeling & inference

Model type and settings	All imaging results were obtained from a single GLM model. We investigated neural correlates using the winning Bayesian jump frequency model. All model predictors were generated with the group mean fitted parameters in order to minimise noise. First level regressors include the onset times for all trials, high pain trials, and low pain trials (duration=0). The all trial regressor was parametrically modulated by model-predicted posterior mean of high pain, the KL divergence between successive posterior distributions on jump probability, and the posterior SD of high pain.<br><br>For second level analysis, both positive and negative T-contrasts were obtained for posterior mean, KL divergence and uncertainty parametric modulators, across all the first level contrast images from all subjects. A group mean brain mask was applied to exclude activations outside the brain. Given that high and low pain are reciprocal in probabilities, a negative contrast of posterior mean of low pain would be equivalent to the posterior mean of high pain. In addition, high and low pain comparisons were done using a subtracting T-contrast between high and low pain trial regressors. We corrected for multiple comparisons with a cluster-wise FDR threshold of p<0.001 for both parametric modulator analyses, reporting only clusters that survived this.
Effect(s) tested	see above

Specify type of analysis: ☒ Whole brain ☐ ROI-based ☐ Both

Statistic type for inference (See Eklund et al. 2016)	cluster-wise
Correction	FDR threshold of p<0.001

Models & analysis

n/a	Involved in the study
☒	☐ Functional and/or effective connectivity
☒	☐ Graph analysis
☒	☐ Multivariate modeling or predictive analysis
